# Mechanical Work and Physiological Responses to Simulated Flat Water Slalom Kayaking

**DOI:** 10.3389/fphys.2019.00260

**Published:** 2019-03-20

**Authors:** Paul W. Macdermid, Aaron Osborne, Stephen R. Stannard

**Affiliations:** ^1^School of Sport and Exercise, College of Health, Massey University, Palmerston North, New Zealand; ^2^Canoe Slalom New Zealand, Auckland, New Zealand

**Keywords:** canoeing and kayaking, intermittent, field testing and monitoring, oxygen deficit, Slalom canoe racing

## Abstract

The purpose of this study was to assess the physical work demand in relation to metrics of force and subsequent physiological response to a simulated flatwater slalom competition. Eight New Zealand team members completed a standard incremental step-test to ascertain power:oxygen consumption relationship. This was followed by a simulated race run where breath-by-breath analysis along with force and power data logged at 50 Hz to determine stroke length, impulse, peak force, time to peak force, and rate of peak force per stroke. Physiological response to negotiating a flatwater slalom course was greater than straight-line paddling (36.89 ± 2.01 vs. 32.17 ± 1.97 ml⋅kg^-1^⋅min^-1^, *p* = 0.0065) at the same power output. Mean power output for the duration of the simulated race (91.63 ± 7.19 s) was 203.8 ± 45.0 W, incurring an oxygen deficit of 1.386 ± 0.541 L⋅min^-1^ translating to an overall anaerobic contribution of 32 ± 18% and aerobic contribution of 68 ± 18%. Moderate to strong relationships between time duration and stroke peak force (*R*^2^ = 0.354, *R*^2^ = 0.485) and rate of peak force development (*R*^2^ = 0.345, *R*^2^ = 0.426) but not for stroke length (*R*^2^ = 0.022, *R*^2^ = 0.012), impulse (*R*^2^ = 0.088, *R*^2^ = 0.097) or time to peak force (*R*^2^ = 0.001, *R*^2^ = 0.0001) for left and right strokes, respectively. The number of propulsive (<0.6 s) strokes outweighed turning/driving (>0.6 s) strokes with a ratio of 94:6%. Longer stroke duration was significantly correlated to greater impulse (*R*^2^ = 0.507, *p* < 0.0001) and time to peak force (*R*^2^ = 0.851, *p* < 0.0001), but a lower rate of force development (*R*^2^ = 0.107, *p* < 0.0001). The results show that a flatwater slalom under simulated race conditions entails initial supra-maximal (anaerobic) work rate with a subsequent transition to one associated with maximal aerobic capacity. Inability to sustain work done and the subsequent decline in peak force and force profile per stroke requires further research regarding strategies to enhance performance.

## Introduction

International Canoe Slalom races are defined by the rules of the International Canoe Federation (ICF), which in turn determines the technical demands and physical environment of competition, and thus the cognitive and physiological stresses on athletes taking part.

Currently, the rules require athletes to race over a predetermined course, changing from race to race, on a section of river involving natural and artificial obstacles, where the length ranges from 200 to 400 m down the center line of the river. Courses involve sequences of two suspended poles referred to as gates (width per gate of 1.2–4.0 m). Each course consists of 18–25 gates, of which six must be negotiated in an upstream direction, and a finish sprint of 15–25 m. Typically, a competent paddler will take 90-s (range: 75–95-s) to complete the course with the fastest total time winning [time penalties are added for hitting (2-s) and missing (50-s) per gate].

Presently, published physiological or physical descriptions of slalom during or following race or simulated race runs are limited (Baker, 1982; Zamparo et al., 2006). These observations include blood lactate values of members of the British team during the 1981 world championships (Baker, 1982). These being 16.2 ± 1.2, 13.1 ± 2.1 and 12.2 ± 1.8 mM for K1M, C1M and K1W categories respectively, taken 5 min following competition. Later studies (Zamparo et al., 2006; Manchado-Gobatto et al., 2014; Messias et al., 2015a,b) report lower post-competition lactate values [mean 7.98 ± 1.6 mM, range: 5.6–10.3 mM in Italian national team members, samples 5 min post-competition (Zamparo et al., 2006)], and suggest a lower intensity, a less capable cohort in the latter study, or a change in emphasis/strategy for success in the sport between studies. Changes in technical regulations may explain this, although there is no information available regarding the work demand or stroke kinetics during competition to corroborate such statements. However, such data is available for flatwater straight-line kayaking, where elite male paddlers typical propulsive stroke have durations of ∼0.44 s, generate peak forces of ∼375 N and an impulse of 109 N⋅s (Baker, 1998; Sperlich and Baker, 2002). Information of this nature taken from slalom paddlers during racing or simulated racing would enhance understanding of the sport whilst being invaluable in the monitoring of athlete development.

Observations of cardiovascular stress during slalom canoeing, peak and mean heart rates are reported to be 184 ± 8 and 173 ± 14 bpm during race simulation (Messias et al., 2015b) and as such, the work rate performed in a slalom race has been considered moderate to vigorous in intensity (Sigmund et al., 2016) with aerobic-anaerobic contribution reported as 24.9% for alactic, 29.9% anaerobic lactic, and 45.2% aerobic (Zamparo et al., 2006).

To our knowledge, there is only one published empirical study indicating metabolic cost during real or simulated competition (Zamparo et al., 2006) who compared all-out flatwater effort with a simulated slalom competition. This work used breath-by-breath gas exchanges and post-exercise blood lactates to estimate aerobic and anaerobic components using a three term model to calculate total metabolic energy (Wilkie, 1981). Results highlighted similarity in aerobic and anaerobic contribution (50–50%, respectively) between conditions even though total energy expenditure was about 30% greater during the all-out flatwater test. Despite the acknowledgment of the importance of power-meters (Sperlich and Klauck, 1992; Messias et al., 2018) and the recent validation of devices capable of recording power output during kayaking (Macdermid and Fink, 2017), no published description of the physical work demand produced by paddlers during course negotiation has occurred.

Thus, the primary aim of this study was to assess the physical work demand and subsequent physiological response to a simulated flatwater slalom competition. Additional aims included a description of stroke kinetics in relation to competition timecourse and stroke length, and a comparison of straight-line and slalom paddling. We hypothesize that: straight-line paddling at the same work rate will trigger smaller physiological response and thus appear more efficient. The simulated race energy distribution will involve maximal demand from both anaerobic during the early stages and aerobic during the latter half but intermittent periods of anaerobic respiration will occur. The ability to generate force and thus work done will decline with time but is dependent on the aim of the stroke. As such, turning strokes will involve greater application of force over prolonged periods resulting in high impulse but low power outputs while propulsive strokes will have low impulse and high power outputs.

## Materials and Methods

### Participants

Eight competitive slalom kayakers who formed part of the New Zealand Slalom development team (mean ± SD, height: 173 ± 4 cm, mass: 65.8 ± 6.0 kg, $\overset{˙}{V}$O_2*max*_ 46.7 ± 4.9 ml⋅kg^-1^⋅min^-1^) were recruited to participate in this study. Prior to their involvement, all participants provided written consent in accordance with the requirements of the University Human Ethics Committee and the Declaration of Helsinki.

### Testing

**FIGURE 1:**
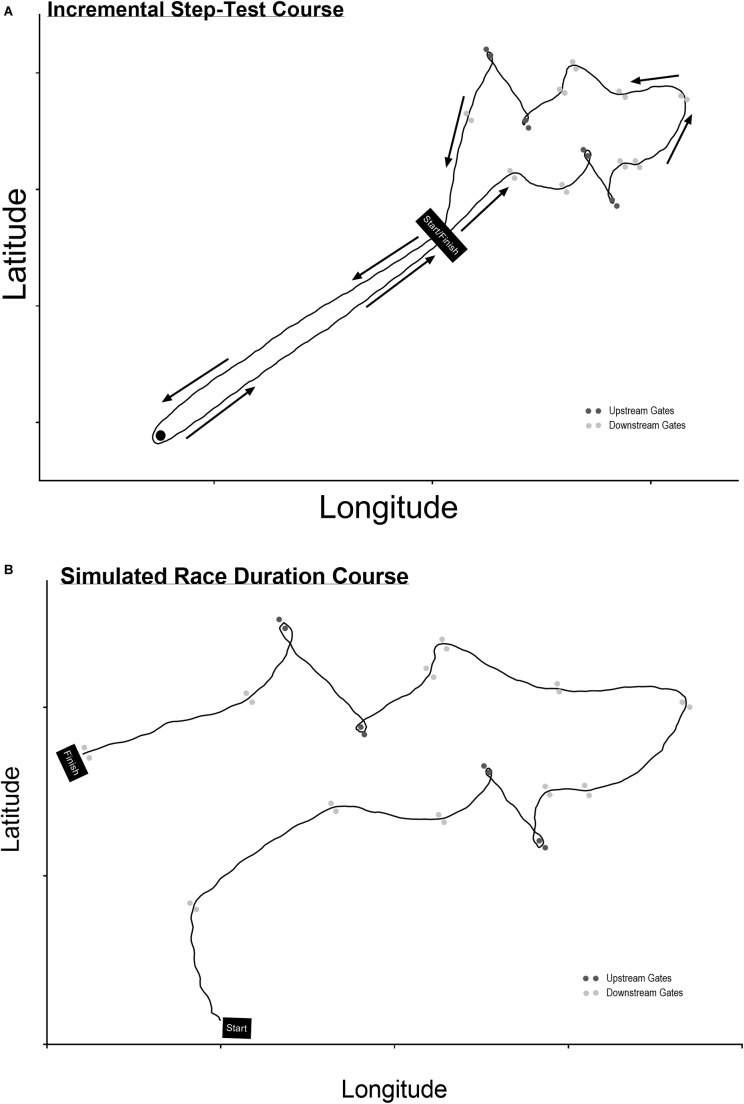
The courses for the **(A)** incremental step-test and **(B)** race duration test. The line is taken from a participant’s high-speed GPS unit.

On arrival at the venue (Centennial Lagoon, Palmerston North, New Zealand) all participants were weighed with and without kayaking clothing and measured (cm). Participants were instructed on the course of gates to be negotiated (Figure 1A,B) in order to mentally plan their route as is typical within competition and training (MacIntyre and Moran, 2007). Once they had deemed sufficient knowledge of the sequence of gates they were fitted with a portable gas analyzer (K42b, COSMED, Rome, Italy).

While participants used their own kayaks, all used the same kayak paddles which included a straight carbon fiber power meter kayak paddle shaft (One Giant Leap, Gisborne, New Zealand) with Naja Maxi elite blades attached (GalaSport, Hrádek, Czechia). Inexperience of some participants (*n* = 3) in using the blades chosen was considered, however all participants were comfortable by the time of any testing. Blade angles in the shaft were adjusted to that preferred and normally used by the participant under normal training and racing conditions.

Following a 5 min warm-up period consisting of straight-line paddling, interspersed with participants preference for turning maneuvres, performed at an intensity ≤ 50 W and acting as a re-familiarization with the test equipment. Participants performed a sub-maximal incremental step-test over a predetermined course (Figure 1A) in order to obtain the Power: $\overset{˙}{V}$O_2_ relationship (Medbo et al., 1988). Data taken from a 5 Hz GPS unit (GPSPORTS, Ltd., Canberra, ACT, Australia) device showed the course length to be 325.3 ± 2.0 m (*n* = 24), while data from the Garmin 920 XT sports watch used to log power data and heart rate provided a length of 291.5 ± 3.8 m (*n* = 24). The course included four upstream gates, 11 downstream gates, inclusive of eight major turning maneuvres. This test consisted of three incremental bouts of exercise, ∼3 min duration at a pre-determined power output, based on fitness, and displayed as average lap power on a portable device (Garmin 920XT) at the front of the kayak cockpit to ensure correct participant work rate. Power output data was logged (1 Hz) throughout the trial and averaged along with $\overset{˙}{V}$O_2_ data (sampled breath-by-breath) over the final 2 min of each stage (Medbo et al., 1988). This enabled an individual power: $\overset{˙}{V}$O_2_ relationship to be formed whilst kayaking and including maneuvres typical in slalom, and where data could be extrapolated to predict O_2_ demand (Medbo et al., 1988; Gastin, 2001) during the competition simulation test described below.

*Supplementary material – time line video of the protocol used* doi: 10.6084/m9.figshare.7376783.

After a 15 min active break which involved 10–12 min of flatwater paddling at an overall intensity deemed light but inclusive of non-monitored short duration high-intensity efforts, followed by a period of rest as per competition race procedures, participants performed one simulated race run (Figure 1B). Throughout, participants wore the automated, portable gas analyzer (breath-by-breath analysis) and used the kayak power meter (data logging 50 Hz). The sequence of gates used was the same as the incremental test previously described (Figure 1A), but differing in the start and finish position. Data taken from 5 Hz GPS unit (GPSPORTS, Ltd., Canberra, ACT, Australia) device showed the course length to be 188.4 ± 2.2 m (*n* = 8).

Participants were instructed to approach the start as per a normal slalom race where athletes work at supra-maximal intensities whilst accelerating to their preferred speed (Zamparo et al., 2006).

The kayak power meter logs force and power data for both left and right shafts separately at 50 Hz. This can be transferred to a standard personal computer as a csv. file and processed using MATLAB R2016a. On a stroke by stroke basis data were analyzed (Macdermid and Fink, 2017) for: (A) Stroke length (s), defined as the time taken from data onset (when the drive side blade triggers a force threshold > 2 N and is a > 10 number points from the end of the previous stroke) to data offset (when the drive side blade triggers a force threshold < 2 N); (B) Impulse (N⋅s), the area under the force curve per stroke; (C) Peak force (N), the maximum force reached during each stroke; (D) Time (s) to peak force, the time from stroke onset to the time of maximum force. Subsequently, the data for the left and right shafts were combined and analyzed for total power output at a sample rate of 50 Hz and averaged over 1 s epochs to coincide with gas analysis data. Additional analysis separated data based on forward propulsive paddling or a turning stroke.

Physiological variables continuously sampled throughout the test included, breath-by-breath expired air in order to calculate $\overset{˙}{V}$O_2_ (L⋅min^-1^) averaged every second. The $\overset{˙}{V}$O_2_ and power output data, used in conjunction with the linear relationship data established via the incremental test was used to estimate O_2_ deficit and subsequently energy system contribution to exercise based on the following calculations:

$$O_{2}demand=mx+b$$

where, *m* is the slope and *b* is y-intercept of the power: $\overset{˙}{V}$O_2_ relationship determined in the incremental test, and *x* is the 1” smoothed average of power output measured during the field test.

$$O_{2}deficit=O_{2}demand-Actual\overset{˙}{V}O_{2}$$

### Statistical Analyses

Data recorded throughout the trials were transmitted to a conventional PC and processed with the Garmin Training Centre, One Giant Leap analysis application^1^ and Cosmed Omnia software provided with the relevant hardware. Descriptive data [mean, standard deviation (SD)] were calculated for all dependant variables. Strokes were categorized as forward (propulsive) or turning via frequency distribution based of stroke length duration. All comparison used a paired *t*-test (GraphPad Prism, Version 7.0) while relationships were determined using linear regression statistics. Significance was set at *p* < 0.05 while classification regarding strength of relationship used Cohen system (Cohen, 1988).

## Results

**FIGURE 2:**
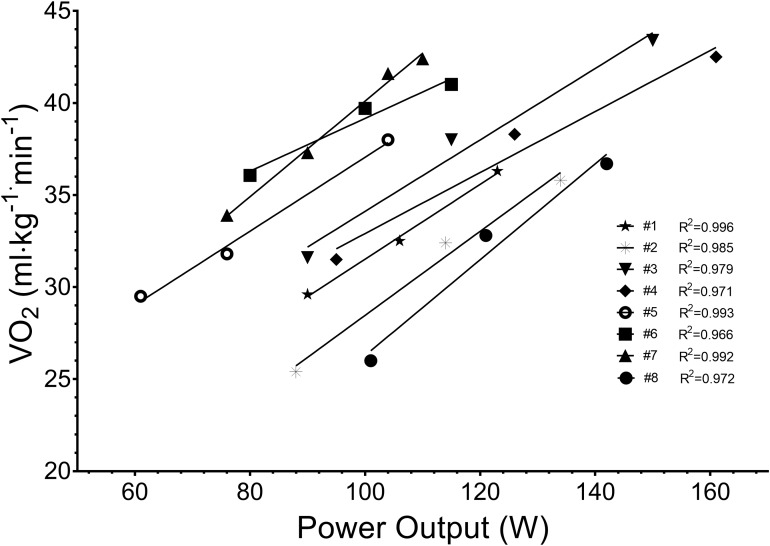
Submaximal field test data for each participant used to determine paddler economy and power: $\overset{˙}{V}$O_2_ relationship.

Participant characteristics obtained from the submaximal and maximal components of the testing are presented in Table 1. The submaximal tests were performed at work rates specific to participants’ state of fitness (Figure 2), and included a straight-line and slalom course component (Figure 1) typical of what occurs during this type of training. Significant difference for $\overset{˙}{V}$O_2_ (32.17 ± 1.97 vs. 36.89 ± 2.01 ml⋅kg^-1^⋅min^-1^, *p* = 0.0065) but not mean power (107.2 ± 16.4 vs. 106.4 ± 15.9 W, *p* = 0.171) were shown when comparing straight-line paddling with the slalom course components respectively.

**Table 1:** Mean ± SD and range for data taken from the submaximal (1–2)-maximal (3–9) component of testing.

	Mean (SD)	Range
(1) $\overset{˙}{V}$O_2_@90W (ml⋅kg^-1^⋅min^-1^)	31.63 (5.09)	23.67–37.73
(2) Economy@90W (W⋅L^-1^⋅min^-1^)	45.23 (8.62)	38.5–64.4
(3) Peak power (W)	606 (169)	418–824
(4) Peak power (W⋅kg^-1^)	8.78 (2.26)	6.26–12.19
(5) W_max_ (W)	203.2 (33.2)	151–303
(6) W_max_ (W⋅kg^-1^)	3.07 (0.69)	2.38–4.32
(7) $\overset{˙}{V}$O_2max_ (L⋅min^-1^)	3.085 (0.328)	2.699–3.559
(8) $\overset{˙}{V}$O_2max_ (ml⋅kg^-1^⋅min^-1^)	46.68 (4.85)	39.3–51.8
(9) Maximum heart rate (bpm)	178 (18)	149–197


**FIGURE 3:**
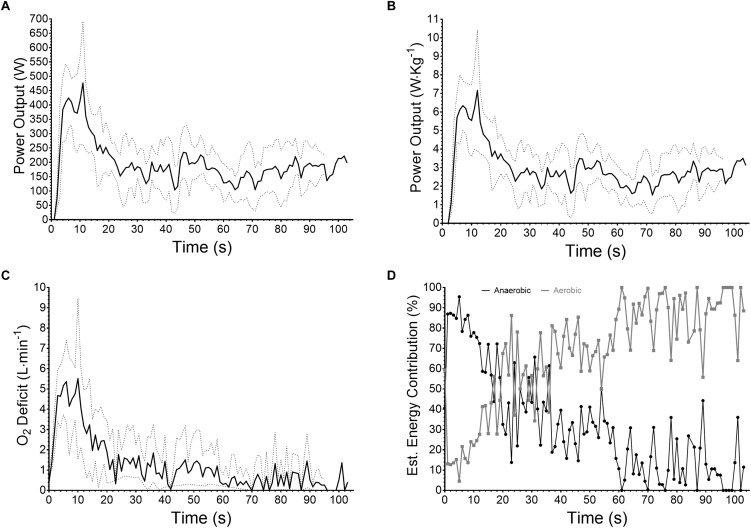
Mean ± SD (*n* = 8) for **(A)** absolute power output (W), **(B)** relative power output (W⋅kg^-1^), **(C)** O_2_ deficit (L⋅min^-1^) and **(D)** mean aerobic-anaerobic contribution to exercise (%).

Mean ± SD time for the simulated race equaled 91.63 ± 7.19 s and this required a mean power output of 203.8 ± 45.0 W or 3.07 ± 0.63 W⋅kg^-1^. Stroke rate was 85.6 ± 58.3 spm of which 94% were forward and 6% turning strokes. Physiological variables associated with these work characteristics for the flatwater slalom equated to rate of oxygen consumption of 2.629 ± 0.498 L⋅min^-1^ (mean ± SD), oxygen deficit 1.386 ± 0.541 L⋅min^-1^, aerobic contribution 68 ± 18%, anaerobic contribution 32 ± 18%, and HR 170 ± 2 bpm. Absolute power output, relative power output, O_2_ deficit and the reliance of anaerobic metabolism decreased over time (Figure 3A–C), while aerobic metabolisms contribution increased (Figure 3D).

**FIGURE 4:**
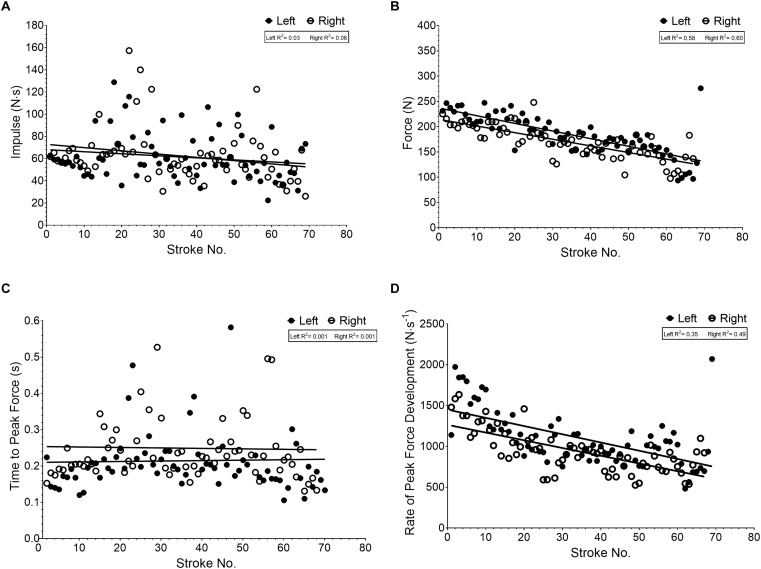
Linear regression for variables of force per left and right stroke [**(A)** impulse, **(B)** peak force per stroke, **(C)** time to peak force, **(D)** rate of peak force development] in relation to stroke number during the flatwater race simulation.

Separate analysis for left and right stroke data presented as mean ± SD for stroke length (0.499 ± 0.184 vs. 0.462 ± 0.213 s, *p* = 0.184), impulse (61.6 ± 21.7 vs. 62.6 ± 23.6 N⋅s, *p* = 0.785), peak force (183.9 ± 39.8 vs. 169.9 ± 33.2 N, *p* = 0.0002), time to peak force (0.215 ± 0.098 vs. 0.249 ± 0.0114 s, *p* = 0.079), and rate of peak force development (1098 ± 339 vs. 942 ± 265 N⋅s^-1^, *p* < 0.0001) provides descriptive kinetics. Linear regression of this data (Figure 4A–D) identified significant negative correlations between stroke number (left and right) and peak force (*R*^2^ = 0.354, *p* < 0.0001 and *R*^2^ = 0.485, *p* < 0.0001, Figure 4B) and rate of peak force development (*R*^2^ = 0.3449, *p* < 0.001 and *R*^2^ = 0.4257, *p* < 0.001, Figure 4D), a weak relationship between impulse (*R*^2^ = 0.088, *p* = 0.014 and *R*^2^ = 0.097, *p* = 0.009, Figure 4A), and no relationships for time to peak force (*R*^2^ = 0.001, *p* = 0.837 and *R*^2^ = 0.0001, *p* = 0.860, Figure 4C) or stroke length (*R*^2^ = 0.022, *p* = 0.220 and *R*^2^ = 0.012, *p* = 0.370). Tests to determine whether the slopes and intercepts (i.e., strategy) are different between participants for strokes indicated a significantly different slope for peak force (*p* < 0.0001) and rate of peak force development (*p* < 0.0001) but not stroke length (*p* = 0.9811), impulse (*p* = 0.1857) or time to peak force (*p* = 0.9402). Where possible intercepts were identified as significantly different (*p* < 0.0001) for stroke length, impulse and time to peak force. Comparisons between performance time and slope (*R*^2^ = 0.4016, *p* = 0.0916; *R*^2^ = 0.4150, *p* = 0.0847) and y-intercept (*R*^2^ = 0.3571, *p* = 0.1177; *R*^2^ = 0.3613, *p* = 0.1150) for peak force and rate of peak force development over the simulated competition run showed moderate-strong relationships, though not significant.

**FIGURE 5:**
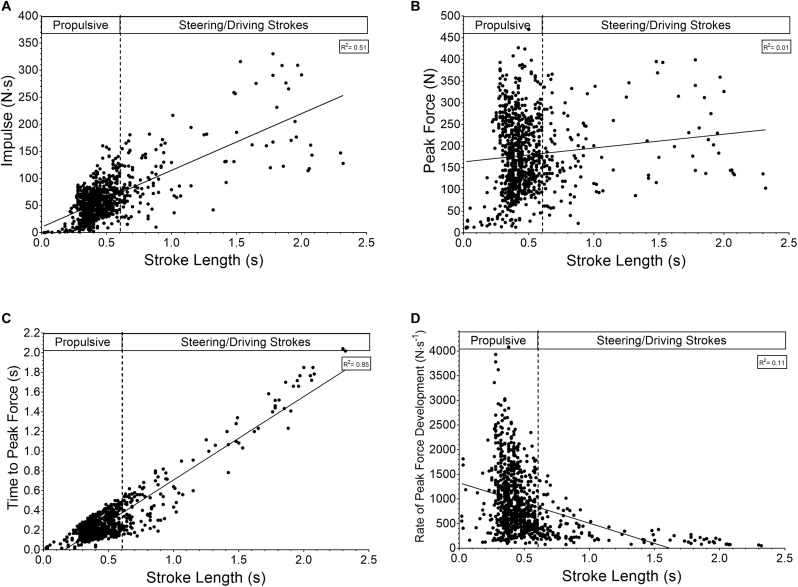
Relationship between stroke length and **(A)** impulse, **(B)** peak force, **(C)** time to peak force, and **(D)** rate of peak force development.

Further analysis of variables of force based around stroke length indicates that strokes of longer duration have greater impulse (*R*^2^ = 0.507, *p* < 0.0001; Figure 5A), vary little with regards to peak force magnitude (*R*^2^ = 0.012, *p* = 0.0009; Figure 5B), take longer to reach peak force (*R*^2^ = 0.851, *p* < 0.0001; Figure 5C), with a lower rate of force development (*R*^2^ = 0.107, *p* < 0.0001; Figure 5D).

## Discussion

This investigation set out to describe the work requirement of a simulated flatwater slalom in the field with reference to physical and physiological demand. In agreement with our hypothesis the main findings were: (a) Paddling in a straight-line with no turning strokes is more efficient than negotiating a course of slalom gates; (b) Forward propulsive strokes are key to simulated race distance on flatwater; (c) Flatwater slalom is dominated by a preponderance to anaerobic respiration in the early stages and during key aspects of competition while high-intensity aerobic respiration dominates overall; (d) Driving/turning stroke force profiles differ to propulsive strokes; (e) Performance is related to the peak force, its rate of development and the corresponding slope of fatigue as the competition progresses.

The introduction of a kayak paddle shaft enabling real time work rate monitoring (Macdermid and Fink, 2017) and subsequently its use in the field, enables valuable monitoring opportunities for athletes, coaches and sports scientist alike. The data presented within Figure 2 and Table 1 display athlete characteristics traditionally gained from a laboratory (Zamparo et al., 2006), which exclude sport specific actions related to steering, balance, and proprioception.

The participants used for this study were all part of the New Zealand development squad from which two members qualified for world cup semi-finals during the year of testing. Even though physiological variables assessed were taken from participants paddling on water, over a pre-determined slalom course, maximal oxygen consumption was similar to those previously reported during laboratory assessments (Pendergast et al., 1979; Vaccaro et al., 1984; Zamparo et al., 2006; Tremblay et al., 2012).

Determining the power:oxygen consumption relationship enabled athlete economy assessment, but primarily was used to estimate energy system contribution as previously reported in other sports (Macdermid and Stannard, 2012). Due to the two basic components of the test used it was possible to compare straight-line paddling with the askew nature of negotiating slalom gates. Paddling at the same work rate over the two components led to a ∼15% increase in physiological demand, i.e., the oxygen consumption over the given epoch at the specific work rate. Care must be taken when interpreting such results as time course may influence the results as the slalom section followed the straight-line component. However, this is unlikely due to the sub-maximal intensities used and is more likely a result of extra physical demand. A result of turning, expressed through longer strokes and a resultant increase in stroke impulse (Figure 5A) typical of turning/driving after periods of deceleration. As such, participants have to work harder to maintain the same power outputs when exercise is intermittent in nature compared to continuous. Future research could use such methods to explore the efficacy of training methods and long-term athlete development concerning physical, technical, and physiological capability along with the ensuing performance. Similarly, changes to equipment design (boats or paddle blades) could be assess physiological response to set work rates and ratified through additional performance testing.

In setting out to simulate a race, participants were asked to approach it with a similar strategy to that which they would typically use. The supra–maximal power outputs presented (Figure 3A,B) implies this occurred, and is associated with a large oxygen deficit (Figure 3C) and thus anaerobic contribution (Figure 3D) to exercise (Gastin, 2001). This is important for a sport that has a large dependency on cognitive function (MacIntyre and Moran, 2007) and where supra-maximal intensities have been shown to impair such performance (Isaacs and Pohlman, 1991) along with physical capabilities (Mendez-Villanueva et al., 2008). As such, paddlers must employ a strategy based on an understanding of physical and physiological capability in order to perform maneuvres demanded by the course set. Failure to judge this correctly will lead to increased chance of incurred time related and/or time-penalty mistakes. It is likely that our participants deemed the technical demand of flatwater slalom easier than a world-class course and as such work rate was higher throughout. However, further research needs to corroborate this, along with optimal pacing strategies in regards to physical and physiological capabilities and the performance outcome. With this in mind, we advocate physical and physiological testing be performed in the athletes normal sport specific environment so data can be used for training prescription and related to real world performance.

Similar duration events, excluding the same level of technicality, have been shown to commence with supra-maximal work rates as shown in Figure 3A,B and likewise settle quickly to a more sustainable level (Craig and Norton, 2001). This level separating the heavy-severe domain of exercise intensity has been termed critical power (Hill, 1993) and relates to an exercise intensity similar to that associated with maximal oxygen consumption capability (Vanhatalo et al., 2007). This supports the oxygen deficit profile presented (Figure 3C) where it drops to a sustainable level between 20 and 30 s and is negligible by 60 s, emphasizing the importance of a large component or requirement for a high aerobic capacity combined with the already established high anaerobic capability.

Variables of force (Impulse, peak force, time to peak force, rate of peak force development, Figure 4) per stroke over the whole simulated race run showed a different pattern (excluding rate of peak force development, Figure 4D) to power output. This data suggests that participants technique with regards to impulse and time to develop peak force remained similar throughout (Figure 4A,C) while the magnitude of peak force decreased linearly (Figure 4B). This could highlight specific aspects in need of developing within this cohort such as an ability to sustain greater peak forces per stroke and can now be monitored through technological advances.

Classifying strokes based on time-force thresholds and using frequency analysis highlights a preponderance for propulsive strokes during flatwater slalom. The relationship between propulsive and steering or driving strokes (Figure 5A–D) suggests that those used to maneuvre the boat require similar peak forces but developed over greater time and thus resulting in greater impulse. These movements are usually associated with reductions in speed or maintaining position in the water. Further research into their nature regarding muscular contraction type, could enhance training methodology within the sport. Additionally, an increase in such movements over a competition run would likely see a competitors average power output decrease suggesting that the ratio of propulsive strokes to steering/driving stroke could be a good indicator of performance or technical capability. Again, it is likely that the 94:6 stroke ratio obtained in this study would differ during actual racing on a white-water course due to the increased demand of steering the boat, but this needs corroborating.

## Conclusion

The results of the present study indicates that a flatwater slalom under simulated race conditions has a power profile dependent upon the course set, but typically evolves around an initial supra-maximal (anaerobic) work rate with a subsequent transition to one associated with maximal aerobic capacity. Turning, accelerating or decelerating maneuvres occurring throughout a competition require high forces generated over a significantly longer time period than forward propulsive strokes, while the ability to generate peak force decreases over the competition period. As such, athletes need to gauge fatigue levels and subsequent effort afforded to propulsion as the competition run progresses in order to sustain turning maneuvres essential to negotiating the course.

### Practical Implications

The following are practical recommendations for athletes and coaches working with slalom kayak athletes:

• The use of a powermeter kayak shaft provides athletes, coaches and support staff quantifiable sport specific data with regards to physical, physiological and technical competency not currently available to slalom kayakers.
• Athlete performance plans need to plan to improve physical, physiological, and technical aspects of paddling that enables maintenance of peak force(s) and its rate of development over a competition run. This requires development of both anaerobic and high intensity aerobic capability combined with aspects of specific (on-water) strength.
• Efficacy of training methodologies, strategy or equipment to attain process-orientated goals focusing on the highlighted demands of the sport can be quantified and monitored.
• Testing and training prescription needs to be specific to individual environment(s) until a greater understanding of the relationship between water/technical demand is formed.

## Author Contributions

PM lead the idea from the beginning to writing of the manuscript. AO participant recruitment and data collection. SS study design and review, writing of manuscript.

## Conflict of Interest Statement

AO was employed by company High Performance Sport NZ and Canoe Slalom NZ. All other authors declare no competing interests.

## Footnotes

1 http://analysis.onegiantleap.co.nz/#/?_k=3rhtaa
